# Talimogene laherparepvec treatment to overcome loco-regional acquired resistance to immune checkpoint blockade in tumor stage IIIB–IV M1c melanoma patients

**DOI:** 10.1007/s00262-020-02487-x

**Published:** 2020-02-12

**Authors:** Anne Fröhlich, Dennis Niebel, Simon Fietz, Eva Egger, Andrea Buchner, Judith Sirokay, Jennifer Landsberg

**Affiliations:** 1 Department of Dermatology and Allergy, University of Bonn, Sigmund-Freud-Str. 25, Venusberg-Campus 1, 53127 Bonn, Germany; 2 Department of Gynecology, University of Bonn, Sigmund-Freud-Str. 25, 53127 Bonn, Germany

**Keywords:** Advanced melanoma, Talimogene laherparepvec, Acquired resistance, Immunotherapy, Targeted therapy

## Abstract

**Background:**

Resistance to immune checkpoint blockade and targeted therapy in melanoma patients is currently one of the major clinical challenges. With the approval of talimogene laherparepvec (T-VEC), oncolytic viruses are now in clinical practice for locally advanced or non-resectable melanoma. Here, we describe the usage of T-VEC in stage IVM1b-M1c melanoma patients, who achieved complete remission or stable disease upon systemic treatment but suffered from a loco-regional recurrence. To our knowledge, there are no case reports so far describing T-VEC as a means to overcome acquired resistance to immune checkpoint blockade or targeted therapy.

**Methods:**

All melanoma patients in our department treated with T-VEC in the period of 2016–2018 were evaluated retrospectively. Data on clinicopathological characteristics, treatment response, and toxicity were analyzed.

**Results:**

Fourteen melanoma patients were treated with T-VEC in our center. Six patients (43%) received T-VEC first-line. In eight patients (57%), T-VEC followed a prior systemic therapy. Three patients with M1b stage and one patient with M1c stage melanoma were treated with T-VEC. These patients suffered from loco-regional progress, whilst distant metastases had regressed during prior systemic treatment. 64% of patients showed a benefit from therapy with T-VEC. The durable response rate was 36%.

**Conclusion:**

T-VEC represents an effective and tolerable treatment option. This is true not only for loco-regionally advanced melanoma patients, but also for patients with stable or regressive systemic metastases who develop loco-regionally acquired resistance upon treatment with immune checkpoint blockade or targeted therapy. A sensible selection of suitable patients seems to be crucial.

## Introduction

Over the last decade, targeted small-molecule inhibitors and checkpoint inhibitors have revolutionized the treatment of advanced melanoma. Targeting BRAF (v-raf murine sarcoma viral oncogene homolog B1), MEK (mitogen-activated protein kinase), the immune checkpoint receptors cytotoxic T-lymphocyte-associated antigen 4 (CTLA-4), and programmed cell death PD-1 has improved the overall survival of melanoma patients. However, in a majority of patients, primary or acquired resistance still limits the durable efficacy of these new therapeutic drugs. Talimogene laherparepvec (T-VEC) is the first oncolytic viral immunotherapy to be approved for patients with locally advanced or non-resectable melanoma (in 2015 in USA and in 2016 in Europe).

T-VEC is a genetically modified herpes simplex virus type 1 designed to selectively replicate in tumor cells. It is attenuated by the deletion of the genes, infectious cell protein (ICP) 34.5 and 47 [1, 2]. In its specific mode of action, T-VEC combines direct oncolytic effects with local and systemic immune-mediated anti-tumoral effects. The release of pro-inflammatory molecules, caused by the viral infection, leads to activation of the innate immune system, the release of interferon gamma, and T-cell infiltration [3]. Deterioration of tumor cells elicits an enhanced liberation of tumor antigens and a priming and increase of tumor-specific T cells. T-VEC is designed to express human granulocyte-macrophage colony-stimulating factor (GM-CSF) and US11 [2, 4]. This cytokine stimulus synergistically enhances the ongoing immune activation, hence promoting a local and systemic anti-tumor immune response, even at non-injected lesions and distant sites [5, 6].

In the primary analysis of the phase III randomized-controlled trial (OPTiM), T-VEC showed only a modest efficacy in visceral metastases, even though T-VEC induces both a local effect through cytolysis and a systemic anti-tumor response through enhancement of antigen presentation and promotion of cytotoxic T-cell responses. A significant improvement of overall survival with T-VEC versus GM-CSF was only observed in the subgroup of melanoma patients with stage IIIB, IIIC, and IVMa1 (41.1 months T-VEC versus 21.5 months GM-CSF, *p* < 0.001) [6, 7]. Therefore, T-VEC has only been approved for advanced loco-regional melanoma excluding patients with lung, brain, bone, or other visceral metastases. Subsequently, results from the final planned analysis of overall survival in the OPTiM trial 3 years after randomization have been published [8]. The final analysis demonstrated a significant improvement of overall survival in the intention-to-treat group. Consistent with the primary analysis, a subgroup analysis showed no significant beneficial effects of T-VEC on overall survival in patients with stage IVM1b/c disease. Recently, clinical studies have investigated therapy with T-VEC in diverse other tumor types and in combination with other systemic treatments, specifically immunotherapy [4, 9, 10]. More widely, there have been substantial advances in the development of oncolytic virotherapy based on diverse DNA and RNA viruses [2].

In clinical studies, strict inclusion and exclusion criteria impose a strong bias compared to the patient population seen in the clinic. More specifically, treatment in the clinical setting includes patients with complex disease history and patients with progressive disease after prior systemic treatments. Analysis of real-life data can thus be informative when considering patient subpopulations of special interest. Yet, published data about use of T-VEC in routine clinical practice are scarce. There are four recent case series from the US that report on use of T-VEC in three single institutions and one two-center retrospective analysis [11–14]. A recent multicenter chart review analyzed data from 27 patients treated with T-VEC in routine clinical practice in Germany [15]. In a current study by the Netherlands Cancer Institute, data from 26 T-VEC treated patients were analyzed based on a prospectively maintained database [16]. Recently, a real-world data study (COSMUS-1) comprising 76 patients in the USA has been published [17]. Together, these published case series and real-world-data studies describe 233 patient and treatment characteristics. However, predictive biomarkers and the identification of patients who can benefit the most from T-VEC are not included. With the growing number of treatment options for advanced melanoma, there is a high clinical need to identify the best treatment strategies and sequences for our patients. In the discussion part, we will, therefore, focus on patient selection criteria based on our single-institution observations as well as the current literature.

This case series aims to characterize the response and follow-up of melanoma patients upon single-agent treatment with talimogene laherparepvec in clinical practice in a single institution in Germany paying a special attention to patients with stable visceral metastases. Although T-VEC has only shown modest efficacy in visceral metastases, we used T-VEC in stage IVM1b-M1c melanoma patients, who had achieved complete remission or stable disease of visceral metastases upon systemic treatment but subsequently suffered from a loco-regional recurrence. To our knowledge, there are no case reports or series so far describing the response of T-VEC as a means to overcome acquired resistance to ICB and/or targeted therapy.

## Materials and methods

We performed a retrospective single-institution review of all melanoma patients who were treated with T-VEC from 2016 to March 2018. Patients were followed up until April 2019. Follow-up was defined as the time period between the last T-VEC application and the last visit to our center, or in the case of deceased patients, the date of death. Patients demographics (age, gender, and ECOG status), clinicopathologic characteristics (melanoma history including prior treatments, disease stage according to the AJCC 7th edition, BRAF status, lactate dehydrogenase LDH, and S100), details of the T-VEC therapy (number of injections, toxicity, and discontinuation), and response to treatment [complete response (CR), partial response (PR), stable disease (SD), and progressive disease (PD)] were reviewed. All injections were performed in cutaneous, subcutaneous, or nodal metastases of melanoma patients at the Skin Cancer Center at the University Hospital in Bonn, Germany. Patients were injected according to the guidelines and recommendations of the manufacturer (≤ 4 ml, 10^6^ plaque-forming units (PFU)/ml on day 1, then after 3 weeks ≤ 4 ml 10^8^ PFU/ml consecutively continued once every 2 weeks. AMGEN, Applied Molecular Genetics, Thousand Oaks, CA, USA). Response to treatment with T-VEC was evaluated clinically in case of cutaneous metastases or using imaging methods such as ultrasound, CT-, or MRI-Scan in the case of subcutaneous, lymph nodes, or visceral metastases. Imaging was conducted before induction of T-VEC and every 12 weeks thereafter. In the analysis of our data, we delineated a subgroup of patients with in-transit metastatic disease and normal baseline LDH levels regardless of tumor stage or line of therapy. We defined these criteria as “low tumor burden”.

## Results

### Patient characteristics and treatment

Fourteen patients with advanced, unresectable melanoma were treated with T-VEC in our center between March 2016 and March 2018. Table 1 gives a detailed overview of the characteristics, treatment, and outcome of each patient. Patients received intralesional T-VEC according to the manufacturer’s recommended dosing. Treatment was continued until no injectable tumor lesions remained, or until intolerable adverse events or progressive disease occurred. Six patients (43%) received T-VEC first-line. In eight patients (57%), therapy with T-VEC followed a prior systemic therapy (75% immunotherapy, 37.5% targeted therapy, 12.5% both, and adjuvant therapy with interferon was not considered as pretreatment). Three patients with M1b stage melanoma and one patient with M1c stage melanoma were treated with T-VEC. These patients suffered from a loco-regional progress, while distant metastases had been stable or regressive during prior systemic treatment. The demographic data are listed in Table 2.

**Table 1:** Patient characteristics, prior treatment, and outcome of *n* = 14 melanoma patients treated with T-VEC

Patient No	1	2	3	4	5	6	7	8	9	10	11	12	13	14
Stage	IIIB	IIIC	IIIC	IIIC	IIIC	IIIC	IIIC	IIIC	IIID	IV M1a	IV M1b	IV M1b	IV M1b	M1c
Baseline LDH ≥ 1.5 × ULN	−	**−**	**−**	**−**	**−**	**−**	**−**	**−**	**+**	**−**	**−**	**++**	**−**	**++**
BRAF mutation status	BRAF V600E	**−**	UN	BRAF V600E	**−**	**−**	BRAF V600D	BRAF V600K	BRAF V600E	**−**	**−**	**−**	**−**	BRAF G469R
Lymph-node metatases present	**−**	**−**	**+**	**+**	**−**	**+**	**+**	**+**	**+**	**+**	**−**	**+**	**−**	**+**
Lymph-node metastases injected (T-VEC)	**−**	**−**	**+**	**−**	**−**	**−**	**−**	**−**	**−**	**−**	**−**	**+**	**−**	**+**
Adjuvant therapy (months)	IFN (2)	0	0	0	0	0	0	0	0	0	IFN (16)	0	0	0
First-line T-VEC	No	Yes	Yes	Yes	Yes	Yes	No	Yes	No	No	No	No	No	No
Prior immunotherapy	No	No	No	No	No	No	Ipi; Nivo	No	No	Pembro	Pembro	Ipi + Nivo; Pembro	Pembro	Ipi + Nivo; Pembrol
Prior targeted therapy	BRAF/MEK	No	No	No	No	No	No	No	BRAF/MEK	No	No	No	No	BRAF/MEK
Number of prior treatment lines	1	0	0	0	0	0	2	0	1	1	1	1	1	2
Number of injections	15	13	12	20	10	7	3	3	3	9	3	1	7	1
Time between prior therapy to T-VEC (weeks)	33	N.A.	N.A.	N.A.	N.A.	N.A.	21	N.A.	0	5	7	3	9	4
Best response	CR	PR	SD	PR	CR	PR	PR	PD	PD	PR	PD	PD	PR	PD
PFS (weeks)	87	83	24	77	101	67	12	0	0	22	0	0	18	0
FU (weeks)	56	N.A.; on treatment	N.A.; Lost to follow-up	16	81	N.A.; on treatment	55	17	43	59	5	20	68	4
Death (PD)								**+**	**+**		**+**	**+**		**+**

**Table 2:** Patient demographics and clinicopathologic characteristics of *n* = 14 melanoma patients treated with T-VEC

	Total (*N* = 14)
Gender, *n* (%)	
Men	6 (43)
Women	8 (57)
Median age, years (min; max)	72.5 (41; 89)
Disease stage (AJCC 2017), *n* (%)	
III B	1 (7)
III C	7 (50)
III D	1 (7)
IVM1a	1 (7)
IVM1b	3 (21)
IVM1c	1 (7)
Line of therapy	
First, *n* (%)	5 (36)
Second or later, *n* (%)	9 (64)
Baseline LDH	240
< ULN	7 (50)
≥ ULN	7 (50)
≥ 1.5 × ULN	3 (21)
Baseline S-100	
< ULN	9 (64)
≥ ULN	5 (36)
BRAF mutation status, *n* (%)	
Mutant	6 (43)
Wildtyp	7 (50)
Unknown	1 (7)
Primary diagnosis, *n* (%)	
Acrolentiginous melanoma	3 (21)
Nodular melanoma	10 (71)
Mucosal melanoma	1 (7)
Tumor thickness, median (min; max)	4.23 (1.0; 11.0)

By the time of database lock (April 2019) on average (median), eight injections were applied (range: 1–41 injections). Two patients were still being treated. In 11 patients (79%), T-VEC was injected in cutaneous metastases. In three patients, T-VEC was injected in subcutaneous or nodal metastases (21%). LDH levels at baseline were elevated in 50% (*n* = 7) of the patients.

### Tolerability/toxicity

T-VEC was in general well tolerated with the toxicity profile as expected from published clinical trials. There were no grade 3 or 4 AEs (according to CTCAE). 89% of the AEs occurred directly after the first injection of T-VEC. Adverse events are shown in Table 3.

**Table 3:** Treatment-related adverse events observed during T-VEC treatment in all patients (*n* = 14)

AE	Grade 1/2, *n* (%)	Grade 3/4, *n* (%)
Any treatment-related AE	9 (64)	0
Chills	6 (43)	0
Pyrexia	5 (36)	0
Fatigue	1 (7)	0
Influenza-like illness	2 (14)	0
Gastrointestinal disorders	5 (36)	0
Injection site reaction	3 (21)	0
Pruritus	1 (7)	0

### Efficacy–response rate

**Fig. 1:**
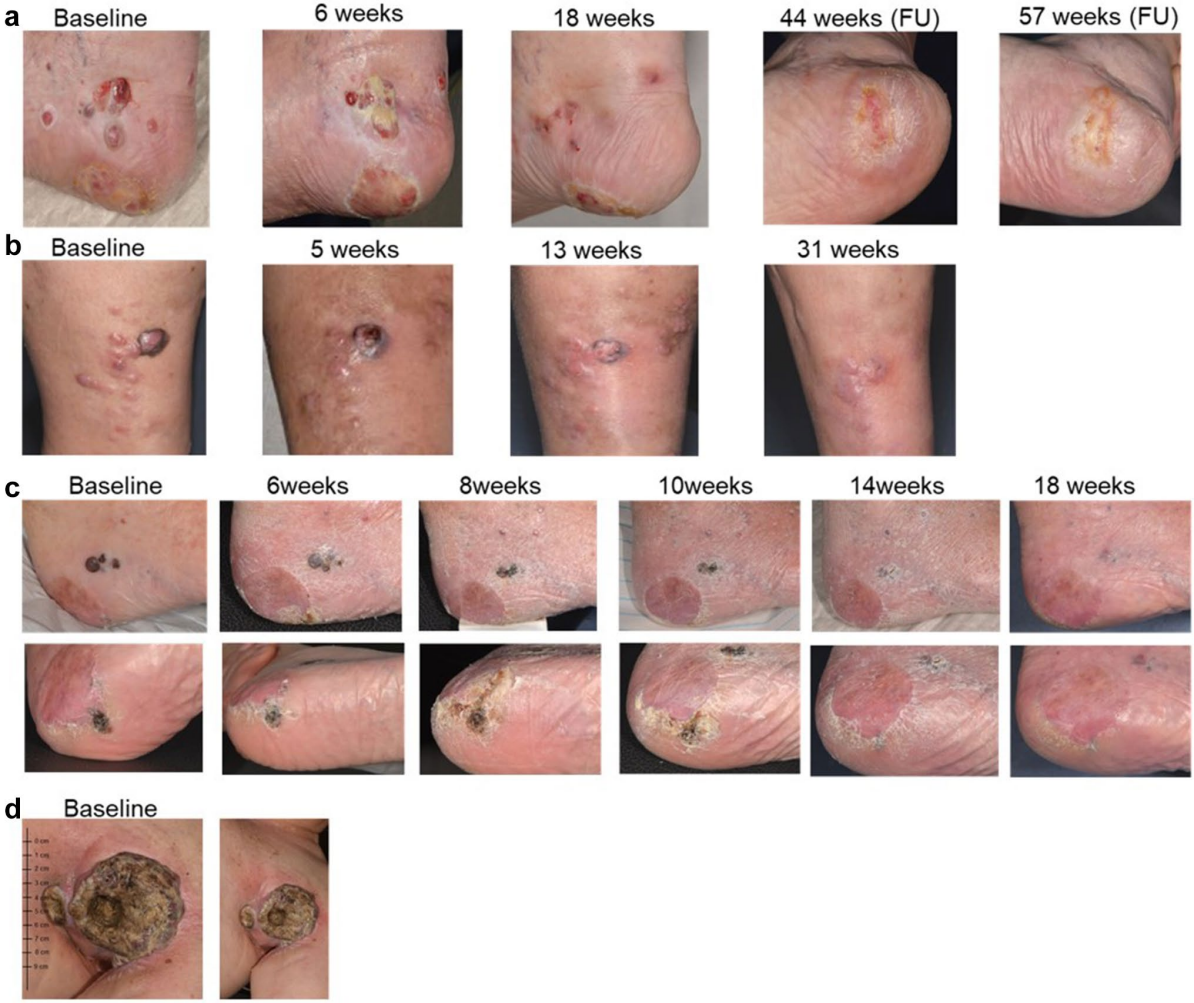
Representative clinical images of four melanoma patients treated with T-VEC over time showing **a** complete response, **b**, **c** partial response, and **d** progressive disease. **d** Baseline image of an ulcerated melanoma metastasis of the left axilla of a melanoma patient (M1c) with prior systemic treatment. Patient received one T-VEC injection and died from rapid progressive disease

In nine patients (64%), a local response to therapy with T-VEC was achieved. Two patients (14%) (No 1 and 5) showed a complete response (CR) and six patients (43%) had a partial response. One patient (No 3) remained at stable disease (7%). Five patients (36%) had a loco-regional or systemic progressive disease. Examples of response and follow-up in four patients treated with T-VEC are shown in Fig. 1.

The median progression-free survival (PFS) was 20 weeks (CI 0; 101). The durable response rate (DRR), defined as complete responses (CR) and partial responses (PR) lasting ≥ 6 months, was 36% (5/14). The five patients (No 1, 2, 4, 5, 6) in our setting showing DRR had stage IIIB and stage IIIC disease. Four of them were treatment-naïve to T-VEC (75%).

A subgroup analysis of patients who received T-VEC treatment first-line (6/14) showed that 83% of these patients had a benefit from treatment with T-VEC. One patient had a complete response. Median PFS was 72 weeks. The DRR in this subgroup was 67%.

**Fig. 2:**
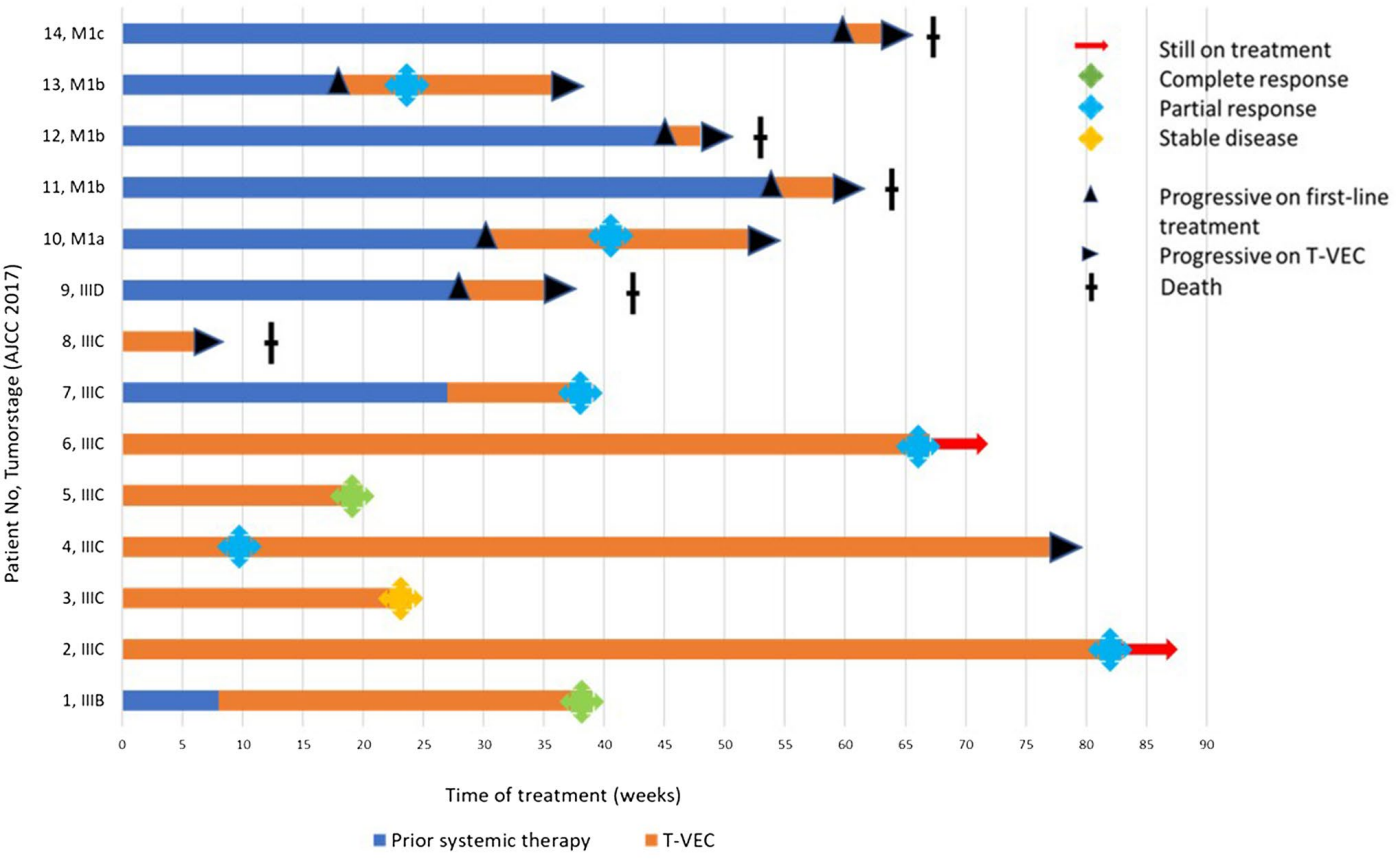
Swimmer plot showing time on prior systemic treatment (in blue) and on T-VEC treatment (in orange) of individual melanoma patients at stage IIIB to M1c (indicated at *y*-axis). Prior systemic treatment includes ICB, targeted therapy, or both. Treatment-free intervals were left out in favor of clarity. Therapeutic responses were defined as best response upon time of data base lock (April 2019). Further systemic therapies following on T-VEC were not considered

Duration of treatment and response on T-VEC are visualized in a swimmer plot (Fig. 2). Outcomes, best response rates, and PFS are summarized in Table 4.

**Table 4:** Best response to T-VEC of all patients and dependent on first-line treatment or with no prior systemic treatment (*n* = 6 patients received T-VEC as first-line treatment; *n* = 8 patients received T-VEC after systemic treatment with immune checkpoint blockade and/or targeted therapy)

	Best response all patients (*N* = 14), *n* (%)	Best response, first-line T-VEC (*N* = 6), *n* (%)	Best response, patients with prior systemic treatment (*N* = 8), *n* (%)
Patients with a response 95% CI			
Complete response	2 (14)	1 (17)	1 (12.5)
Partial response	6 (43)	3 (50)	3 (37.5)
Stable disease	1 (7)	1 (17)	0 (0)
Progressive disease	5 (36)	1 (17)	4 (50)
Overall response rate	9 (64)	5 (83)	4 (50)
Durable response rate	5 (36)	4 (67)	1 (12.5)
Progression-free survival, median (weeks)	20	72	10
Death	5 (36)	1 (17)	4 (50)

Eight patients (57%) received T-VEC following a prior systemic therapy. Six patients had received a prior treatment with anti-PD1-therapy (No 7, 10–14). The median period of time between two therapies was 6 weeks (CI 3; 21). In five patients (No 10–14), treatment with T-VEC almost continuously followed prior anti-PD1-therapy (CI 3; 9 weeks). Two patients were treated with a BRAF and MEK inhibitor prior T-VEC therapy (No 1, 9). One patient (No 14) with tumor stage M1c had received anti-PD1 followed by BRAF inhibition with vemurafenib, and a third line combined anti-PD1 and anti-CTLA4 immunotherapy before treatment with T-VEC was induced. The median PFS in patients treated with T-VEC second-line was 10 weeks (CI 0; 87), compared to 72 weeks (CI 6; 101) in the first-line treated patients.

One patient with in-transit metastatic melanoma, stage IIIB (patient No 1) received T-VEC after prior targeted therapy with BRAF/MEK inhibitor and obtained a long-lasting, complete response. Another patient (patient No 10) with M1a stage melanoma received prior anti-PD1-therapy, which led to a regression of lymph-node metastases, whereas in-transit metastases showed loco-regional resistance to immunotherapy. Here, treatment with T-VEC achieved a partial response in in-transit metastases. A stage IIIC melanoma patient (No 8) received T-VEC following two lines of prior immunotherapy. These three patients who responded to T-VEC all showed low tumor burden, as defined by in-transit metastases and normal LDH levels. Patient No 9, with irresectable tumor stage IIID melanoma, received T-VEC following a targeted therapy with BRAF/MEK inhibition. The patient suffered from a large tumor, increased LDH levels, and did not respond to T-VEC therapy.

Four patients with advanced melanoma, stage M1b-M1c, who had received one or more prior systemic therapies were treated with T-VEC (patients No 11–14). These patients suffered from a loco-regional progression, while distant metastases had been stable or regressive during prior systemic treatment.

Three of these patients (No 11, 12, 14) did not respond to T-VEC and had a rapid progressive systemic disease. Hence, T-VEC could only be applied once and three times, respectively. These patients suffered from a high loco-regional tumor burden with elevated S-100 and LDH levels at baseline.

One patient with advanced melanoma (patient No 13) showed a partial response from treatment with T-VEC. The patient had received prior anti-PD1 treatment with pembrolizumab in M1b stage melanoma, upon which a complete response of the known lung metastases was documented by repeated CT-scans every 12 weeks. In the course of time, the patient developed multiple cutaneous in-transit metastases of the lower extremity without any systemic progression. LDH levels were within normal range and tumor burden as per our definition was low. Cutaneous metastases were treated with seven cycles of T-VEC and lesions showed a partial response for 18 weeks before the patient developed progressive disease with new pulmonal metastases. Immunotherapy with anti-PD-1 was re-induced and achieved a complete response.

In total, three patients in our center (21%) had baseline LDH levels ≥ 1.5-fold above upper limit normal (ULN); all of them had received a prior systemic treatment. None of these patients responded to T-VEC.

Our results show that treatment with T-VEC achieved a response in patients with low tumor burden, having limited in-transit metastases and normal LDH levels at baseline. This held true even for patients with prior systemic therapy and for a patient with M1b melanoma.

## Discussion

We present a retrospective single-institution case series of 14 patients who were treated with T-VEC for locally advanced metastatic melanoma. In contrast to recently published case series, in our study, four stage IVM1b-M1c patients, who developed loco-regional progression, while distant metastases showed complete remission upon ICB or/and targeted therapy, received T-VEC treatment. A recent case series from USA reported about 26 melanoma patients in stage IIIB–IVM1a and one patient in stage IVM1c receiving T-VEC in a single institution [12]. This work does not report detailed information about the IVM1c melanoma patient. A recent multicenter chart review analyzed data from 27 T-VEC treated melanoma patients and aimed to characterize the first patients in Germany treated with T-VEC [15]. This trial included seven patients who had received prior immunotherapy, but all these patients had stage IIIB–IVM1a disease. A third recently published case series, again from the US, reviewed ten patients in a participating center of the Masterkey-256 study, who did not meet the eligibility criteria, but were treated off-label with T-VEC plus checkpoint inhibitors [11]. A current study based on a prospectively maintained database from the Netherlands Cancer Institute showed high complete and overall response rates in 26 stage IIIB/C in-transit melanoma patients. Twenty-three patients were treatment-naïve [16]. The COSMUS-1 study investigated T-VEC treatment in the clinical practice setting in 78 patients in the USA, including 43.4% patients who received checkpoint inhibitors before T-VEC treatment or in combination and 30 patients with tumor stage IVM1b/c disease [17].

The overall response rate (ORR) in our study was 64%. The ORR is similar to the observed ORR of 56.5% in the US case series published by Sun et al. [11] but significantly higher than the observed ORR in the primary analysis of the phase III OPTiM trial (26.4%) [6, 12] and the final analysis of the OPTiM data (31.5%) [8]. The difference in ORR between our study and the OPTiM trial is mainly related to the higher number of stage IVM1b/c melanoma stages (43 in the primary analysis [6], 45 in the final analysis [8] vs 29% in our study). Andtbacka et al. stated that T-VEC efficacy was most pronounced in patients with stage IIIB–IVM1a disease and in treatment-naïve patients. The DRR in our subgroup of first-line patients was 67%. This is a considerably high response rate compared to the primary subgroup analysis in the OPTiM trial which produced a DRR of 24% in first-line patients. The recently published final planned OPTiM trial analysis confirmed the results of the primary analysis, showing significant beneficial effects of T-VEC on overall survival in the subgroup of patients receiving T-VEC first-line [8]. The recent study of Franke et al. demonstrated a high ORR of 88.5% and best CR rates of 61.5% in a mainly treatment-naïve population of IIIB/IIIC in-transit melanoma patients (median duration of response not yet reached) [16]. The authors suggested patients with early metastatic disease stage IIIC-M1a and low tumor burden to be the subgroup of patients who are most likely to benefit from T-VEC. This applied to patients with in-transit metastatic disease, as patients with lymph-node involvement were excluded from the trial. Our data support the assumption that patients with low tumor burden, indicated by in-transit metastatic disease and by normal LDH values, are a highly favorable subgroup for treatment with T-VEC. The primary effect of oncolytic therapy is mediated by induction of a local anti-tumor response, which is more likely to control a locally limited tumor disease. However, it is unclear if the favorable effects of T-VEC in early stage melanoma are based on better activity in early stage disease, or merely reflect the natural history of melanoma. As demonstrated in the current AJCC cancer staging manual, the 5-year melanoma-specific survival rate significantly ranges according to disease subgroups, e.g. from 83% in patients with stage IIIB disease to 32% for those with stage IIID disease [18].

In our study, only patients with normal LDH levels responded to T-VEC therapy. The OPTiM study excluded patients with baseline serum lactate dehydrogenase (LDH) ≥ 1.5 × ULN. The enzyme LDH is a serological biomarker in melanoma and one of the strongest prognostic indicators found to be correlated with tumor burden [19, 20]. The exclusion of patients with baseline serum LDH ≥ 1.5 × ULN leads to a strong bias towards patients with lower tumor burden. In our cases, three patients (21%) had baseline LDH levels ≥ 1.5 × ULN, with all of them having received a prior systemic treatment. None of these patients responded to T-VEC. In these three patients, staging imaging preceding induction of T-VEC had demonstrated visceral disease to be stable or absent and progressive disease was limited to in-transit or lymph-node metastases. Yet, we can assume that increased LDH levels in these patients not only reflected high tumor load, but, as a marker for active progressive disease, indicated the rapid progression of the melanoma. Patients with complete or partial response to T-VEC (≥ 6 months) all showed baseline LDH levels ≤ 1.5-fold above limit. This is in line with the assumption that therapy with T-VEC is particularly effective in patients with low tumor load. This criterion is met more often in the absence of visceral disease which applies to the approved indication of the drug and our definition of low tumor burden. However, this is also true for other systemic melanoma therapies as low tumor burden is a favorable prognostic factor for overall survival and overall response rate to systemic therapy in general. For targeted therapy with BRAF/MEK inhibitors, there is a known correlation between baseline tumor burden, response rate, and progression-free survival [21, 22]. Another recent study investigated the connection between T-cell invigoration, tumor burden, and anti-PD1 response in immunotherapy with pembrolizumab [23].

Baseline tumor burden has been demonstrated to be a predictive factor in diverse malignancies, including melanoma [24]. In T-VEC-treated patients included in the OPTiM trial, achievement of CR showed a significant negative association with baseline tumor burden [25]. Based on a multivariate analysis in this trial, the cut-off for baseline tumor burden was > 14.5 cm [8]. The two-center retrospective analysis including 40 patients in USA reported increased overall survival and PFS in patients with smaller tumors [14]. The retrospective single-center analysis in 27 patients demonstrated decreased efficacy of T-VEC with increasing lesion size [13]. However, a general definition for tumor burden does not exist. Depending on the study design, tumor burden has been defined in several ways, including number of metastases, tumor diameter, or volume [24]. Kaufman et al. stated that tumor burden was prognostic for overall survival and achieving a complete response in the OPTiM trial. Hereby, multivariate analysis showed a significant association of tumor burden and tumor stage (IIIB–IVM1a vs. IVM1b/c) and treatment line (fist line vs. latter line) [25]. This held true for the majority of our patients as well. However, in our case series, we particularly focus on stage IV melanoma patients who received a loco-regional response to T-VEC after having received a systemic response to immune checkpoint blockade. These patients did not fulfill the criteria of tumor stage IIIB–IVM1a and T-VEC treatment first-line. However, these patients suffered only from in-transit metastatic disease and showed normal baseline LDH levels. In our study, no prospective quantification of tumor volume, diameter, or RECIST evaluation was performed, limiting our ability to define a quantitative cut-off for tumor burden. We, therefore, refer to “low tumor burden” as in-transit metastatic disease with normal baseline LDH to differentiate this subgroup of patients irrespective of tumor stage or line of therapy.

In general, the criteria of early metastatic melanoma and low tumor burden might more often be met in treatment-naïve patients. Efficacy in patients treated with T-VEC first-line is suggested to be most pronounced [6]. However, data on the influence of prior systemic treatment are scarce.

Two case series highlight the clinical efficacy of T-VEC after progression on multiple previous therapies [26, 27]. In accordance with these publications, in our center, four out of eight patients profited from T-VEC after ICB or/and targeted therapy. In contrast to the afore mentioned case series, we present not only data on stage IIIB/C and IVM1a pre-treated melanoma patients, but also four stage M1b-M1c patients who achieved complete remission of visceral metastases with ICB or/and targeted therapy and developed loco-regional progression. In the COSMUS-1 observational study, 21 patients received immune checkpoint blockade prior T-VEC treatment and 3 out of 21 showed complete response [17]. The study included 30 patients with tumor stage IVM1b/c disease. However, the authors did not investigate in depth which of those patients benefited from treatment with T-VEC. Six patients treated in our center had received a prior therapy with anti-PD1-therapy, two patients responded to T-VEC. In our institution, three patients had received a prior treatment with targeted therapy (BRAF/MEK inhibitors), and one patient profited from treatment with T-VEC.

It has been suggested that previous treatment exposure may have an effect on outcome to different therapeutic agents [28]. In the OPTiM trial, patient enrolment took place from May 2009 to July 2011. This indicates that options for prior therapy were limited compared to contemporary state of the art therapy. The impact of previous systemic therapy on response to T-VEC thus remains an open question [26]. A retrospective analysis of 27 melanoma patients treated with T-VEV found a decreased ORR for pre-treated patients without describing details of these patients [13]. Our data suggest that some patients’ clinical course may benefit from prior therapy with T-VEC and may even pave the way to long-term response. In our center, all patients that profited from T-VEC as the second-line treatment had low tumor burden, defined as disease limited to in-transit metastases and normal LDH levels. Patients with high tumor burden and a rapid tumor progression did not respond to second-line T-VEC treatment. Even though LDH level is not reported or not correlated to clinical response in other case series with pre-treated melanoma patients who received T-VEC as second-line treatment [12, 15, 26, 27], we suggest that elevated LDH level can serve as a useful indicator. A sensible selection of suitable patients seems to be crucial and high medical need patients seem to require more aggressive therapeutic intervention.

The combination of oncolytic virotherapy and immunotherapy is a promising approach. As described in the patient characteristics, one patient with M1b disease (No 13) profited from T-VEC treatment in in-transit metastases, which had occurred under prior treatment with anti-PD-1 therapy. The patient showed a low tumor burden with normal LDH levels and in-transit metastases. We conjecture that T-VEC injections provoked an anti-tumor response, which helped to overcome acquired resistance to immunotherapy. It is remarkable that upon progressive disease with new lung metastases after 18 weeks on T-VEC, re-induced anti-PD-1 therapy yielded a complete response. It is probable that in this case, T-VEC provided synergistic efficacy in reinvigorating the exhausted immune response. The combination of T-VEC and anti-PD1-therapy has currently been tested in a phase Ib trial (Masterkey-256) [9]. Twenty-one patients with advanced melanoma were treated with T-VEC followed by combination with pembrolizumab. The objective response rate was 62% with a complete response rate of 33%. The authors suggested that oncolytic therapy may improve the efficacy of immunotherapy with anti-PD1-antibodies mainly by affecting the tumor microenvironment [9]. Sun et al. recently published a case series of ten patients who were treated off-label with T-VEC plus checkpoint inhibitors [11]. The surveyed data support the idea that combination of checkpoint inhibitors with T-VEC may provide a synergistic effect. Outcome was even superior in comparison to published studies on similar therapeutic regimes, although validity might be limited by the small number of patients. To our knowledge, there is no clinical trial investigating the combination of targeted therapy and T-VEC at this point. A recently published case report describes the clinical course of a heavily pre-treated 71-year-old patient with stage IIIB disease who profited from T-VEC. Prior therapies included GM-CSF, vemurafenib, dabrafenib, trametinib, ipilimumab, and pembrolizumab [26].

We are aware of the limitations of our study. Response to treatment with T-VEC was evaluated in some patients by clinical and/or sonographic measurement of the injected lesions and might, therefore, in part vary depending on the investigators’ experience. Limited by the retrospective nature of our study, we decided against the definition of a quantitative measurement of tumor burden. Due to the small number of patients, the heterogeneity of our cohort, the retrospective setting, as well as unknown potential confounding variables, the possibility to draw generalizable conclusions from our case series is limited. In summary, we observed a positive response in 9/14 T-VEC-treated patients with loco-regionally advanced melanoma. As stated in previous trials, there was a trend towards greater therapeutic benefit in patients with low tumor burden and stage IIIB-M1a disease. We observed a better DRR in the first-line patients in comparison to the approval trial. We observed a partial response from T-VEC in a patient who developed loco-regional resistance to anti-PD1 therapy. Moreover, our data suggest that patients who have previously received systemic therapy can still profit from therapy with T-VEC and, in some cases, even achieve long-term responses. This was true for patients with low tumor burden, characterized by normal baseline LDH levels and in-transit metastatic disease, which can serve as an identification mark for characterizing this group of patients. We, therefore, suggest that tumor burden rather than tumor stage is crucial for the identification of patients who are most likely to benefit from treatment with T-VEC.

We propose that T-VEC treatment is an attractive option not only for pre-treated stage IIIB/C and IVM1a patients but also for patients with stable visceral metastases who acquired loco-regional resistance to ICB and/or targeted therapy. A sensible selection of suitable patients seems to be crucial. Moreover, the combination of T-VEC with other systemic therapies, specifically immunotherapy, seems to be a promising prospect.

## Abbreviations

BRAF: V-raf murine sarcoma viral oncogene homolog B1
CR: Complete response
DRR: Durable response rate
ICB: Immune checkpoint blockade
ORR: Overall response rate
PD: Progressive disease
PR: Partial response
SD: Stable disease
T-VEC: Talimogene laherparepvec
ULN: Above upper limit normal
US 11: Accessory factor US11
